# FourFold Asthma Study (FAST): a study protocol for a randomised controlled trial evaluating the clinical cost-effectiveness of temporarily quadrupling the dose of inhaled steroid to prevent asthma exacerbations

**DOI:** 10.1186/s13063-016-1608-6

**Published:** 2016-10-13

**Authors:** Andrew Skeggs, Tricia McKeever, Lelia Duley, Eleanor Mitchell, Lucy Bradshaw, Kevin Mortimer, Samantha Walker, Steve Parrott, Andrew Wilson, Ian Pavord, Chris Brightling, Mike Thomas, David Price, Graham Devereux, Bernard Higgins, Tim Harrison, Rebecca Haydock

**Affiliations:** 1 Nottingham Clinical Trials Unit (NCTU), Nottingham Health Science Partners, C Floor, South Block, Queen’s Medical Centre, Nottingham, NG7 2UH UK; 2 Division of Epidemiology and Public Health, Clinical Sciences Building, University of Nottingham, Nottingham, NG5 1PB UK; 3 Liverpool School of Tropical Medicine, Pembroke Place, Liverpool, L3 5QA UK; 4 Asthma UK, 18 Mansell Street, London, E1 8AA UK; 5 Department of Health Sciences, Alcuin C Block, Alcuin College, University of York, York, Y010 5DD UK; 6 Department of Health Sciences, University of Leicester, Leicester, LE1 6TP UK; 7 NDM Research Building, Nuffield Department of Medicine, University of Oxford, Old Road Campus, Roosevelt Drive, Oxford, OX3 7FZ UK; 8 Respiratory BRU, Glenfield Hospital, University of Leicester, Leicester, UK; 9 University of Southampton, Aldermoor Health Centre, Aldermoor Close, Southampton, SO16 5ST UK; 10 Chest Clinic C, Aberdeen Royal Infirmary, Aberdeen, AB25 2ZN UK; 11 Centre of Academic Primary Care, Division of Applied Health Sciences, University of Aberdeen, Polwarth Building, Aberdeen, AB25 2ZD UK; 12 Respiratory Medicine, Cardiothoracic services, The Newcastle upon Tyne Hospitals NHS Foundation Trust, Freeman Hospital, Newcastle upon Tyne, NE7 7DN UK; 13 Nottingham Respiratory Research Unit, Clinical Sciences Building, City Hospital Campus, University of Nottingham, Nottingham, NG5 1PB UK

**Keywords:** Asthma, Exacerbation, Self-management plan, Inhaled corticosteroids, Oral corticosteroids Randomised controlled trial, Fourfold, Protocol, Primary care

## Abstract

**Background:**

Asthma is one of the commonest chronic diseases in the UK. Acute exacerbations of asthma are unpredictable, disruptive and frightening. They cause considerable morbidity and account for a large component of the health service costs of asthma. The widespread use of an asthma self-management plan, designed to encourage disease monitoring and timely intervention, can reduce exacerbations and is, therefore, recommended for all patients with asthma. Unfortunately, the majority of patients are not provided with such a plan. There are a variety of reasons for this but uncertainty about what to include in the plan when asthma control is deteriorating, but before the need for orally administered corticosteroids, is a contributing factor.

The aim of this trial is to determine whether an asthma self-management plan, which includes a temporary quadrupling of the dose of inhaled corticosteroid when asthma control starts to deteriorate, reduces asthma exacerbations requiring orally administered corticosteroids or unscheduled health care consultation for asthma.

**Methods:**

A multicentre, pragmatic, randomised trial in adults aged over 16 years with a clinical diagnosis of asthma, treated with a licensed dose of inhaled corticosteroid and at least one exacerbation in the previous 12 months requiring treatment with systemic corticosteroids. Participants will be randomised to either a self-management plan, which includes a temporary (maximum of 14 days) fourfold increase in inhaled corticosteroid or the same plan without an increase in inhaled corticosteroid. Participants will be followed up at 6 and 12 months and will attend the clinic for an additional visit if their asthma control deteriorates. The primary outcome is time to first asthma exacerbation, defined as the need for systemic corticosteroids and/or unscheduled health care consultation for asthma. The estimated sample size is 1800 participants.

**Discussion:**

The FAST trial is an independent study that has been prioritised and commissioned by the National Institute for Health Research (NIHR) in the United Kingdom. It will provide high-quality evidence to inform clinical decision-making on the role of an asthma self-management plan, which includes a temporary fourfold increase of inhaled corticosteroid, when asthma control starts to deteriorate.

The first participant was randomised on 17th May 2013 and recruitment will close on 31 January 2016 with the last patient last visit taking place in January 2017.

**Trial registration:**

ISRCTN: 15441965, registered on 25 April 2013.

**Electronic supplementary material:**

The online version of this article (doi:10.1186/s13063-016-1608-6) contains supplementary material, which is available to authorized users.

## Background

Asthma is one of the commonest chronic diseases in the UK. Acute exacerbations or attacks of asthma cause considerable morbidity and account for a large component of the costs of asthma.

Self-management plans have been shown to reduce exacerbations requiring orally administered corticosteroids and emergency health care utilisation [1]. Unfortunately, the majority of patients with asthma are not provided with a written self-management plan [2] and only 23 % of patients dying from asthma in the UK were known to have been given a written self-management plan [3]. Although there are a variety of reasons for this, confusion about what to include in the plan when asthma control is deteriorating, but before the need for orally administered corticosteroids, is one important limiting factor.

Two large, randomised, double-blind, placebo-controlled clinical trials have found no benefit from doubling the dose of a patient’s usual inhaled corticosteroid [4] or doubling the dose of inhaled budesonide [5] when asthma control starts to deteriorate. However, other studies have suggested that a fourfold increase [6], a fivefold increase [7] or 2 mg inhaled fluticasone propionate added to current therapy [8] can prevent the progression of an exacerbation.

The aim of this trial is to determine whether an asthma self-management plan, which includes a temporary quadrupling of the dose of inhaled corticosteroid when asthma control starts to deteriorate, reduces asthma exacerbations requiring orally administered corticosteroids or unscheduled health care consultation for asthma.

### Objectives

Overall we will determine the clinical and cost-effectiveness of an asthma self-management plan, which includes a temporary quadrupling of the dose of inhaled corticosteroid when asthma control starts to deteriorate, at preventing an asthma exacerbation which is defined as: the need for systemic corticosteroids and/or unscheduled health care consultation for asthma (i.e. reaching zone 3 or 4 of the Asthma UK self-management plan).

The primary objective it to determine whether the proposed asthma self-management plan reduces asthma exacerbations requiring orally administered corticosteroids or unscheduled health care consultation for asthma.

The secondary objectives are to (1) determine whether the proposed asthma self-management plan reduces the deterioration in asthma control, and (2) to determine if the proposed asthma self-management plan is cost-effective to the NHS and society overall.

## Methods/design

This is a multicentre, pragmatic, randomised trial comparing the clinical and cost-effectiveness of a self-management plan, which includes a temporary fourfold increase in inhaled corticosteroid with the same plan without an increase in inhaled corticosteroid.

**Fig. 1:**
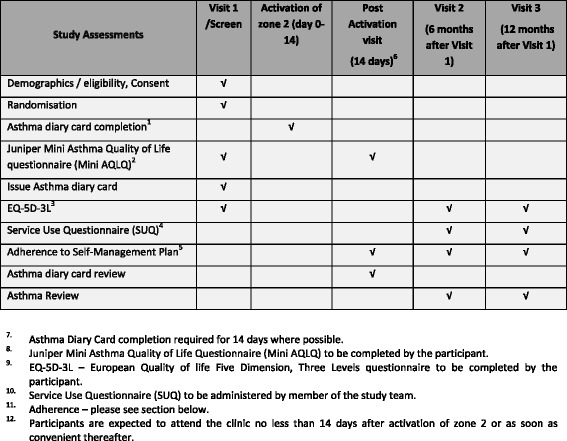
Timetable of study assessments

Flow of participants through the trial is summarised in Fig. 1.

## Participants, interventions and outcomes

### Setting

Potential participants will be recruited from both primary and secondary care across England and Scotland and through local advertising (self-referral). We envisage that the majority of participants (approximately 80 %) will be recruited within primary care.

For primary care we plan to recruit from general practitioner (GP) practices across England and Scotland in conjunction with the Primary Care Research Network (PCRN/SPCRN). The model of recruitment will depend on the models of delivery within the differing PCRN areas. The lead PCRN based within the chief investigator’s region (East Midlands and South Yorkshire PCRN) will be responsible for facilitating the set-up of the study within primary care and will liaise with the relevant local PCRNs based within the collaborating centres’ region. Local PCRNs will liaise directly with GP practices who will act as Participant Identification Centres (PICs).

At secondary care sites participants will be identified from patients attending respiratory outpatient appointments at the individual NHS Trust recruiting centres. The first contact will be made by the patient’s clinician or a member of their immediate care team within the clinic. They will be given a brief overview of the study and a Participant Information Sheet (PIS) will be provided. Sites were chosen on the basis of a proven track record of recruiting into previous asthma trials.

### Eligibility

#### Inclusion criteria

- Male or female patients aged 16 years and over
- Clinician-diagnosed asthma treated with a licensed dose of inhaled corticosteroid (i.e. steps 2 to 4 of the British Thoracic Society/Scottish Intercollegiate Guidelines Network (BTS/SIGN) guidelines)
- One or more asthma exacerbation in the last 12 months requiring treatment with systemic corticosteroids

#### Exclusion criteria

- A history more in keeping with smoking-related chronic obstructive pulmonary disease (COPD) (smoked more than 20 pack years, without evidence of significant reversibility and an absence of eosinophilia)
- On maintenance orally administered corticosteroids (i.e. step 5 of the BTS/SIGN guidelines)
- Using a combination inhaler for both maintenance and relief treatment
- Experienced an asthma exacerbation within 4 weeks of randomisation
- Pregnant women, lactating women or women who are planning to become pregnant

### Interventions

Participants will be randomised equally to one of two asthma self-management plans developed from the Asthma UK plan in use at the time (Appendix 1). Zones 1, 3 and 4 are identical and zone 2 includes the current area of uncertainty and the research question under investigation (Appendix 2).

At randomisation, participants will be provided with instructions on how to follow their allocated asthma self-management plan and given guidance on how to complete the Asthma Diary Card for 14 days when their asthma deteriorates.

On reaching zone 2 of the plan the ‘modified group’ will be advised to increase their bronchodilators and quadruple their inhaled corticosteroid dose for 7 to 14 days (Appendix 3 and 4) and the ‘usual care group’ will be advised to increase their bronchodilator medication only.

Assessment of adherence with the two self-management plans will include a review of the Asthma Diary Card and specific questions about whether, and how, participants changed their inhaled treatment since activating zone 2 of the self-management plan. Adherence will be classified as:

- In the modified (quadrupling) group:
  - ∘ poor – no or minimal change in medication
  - ∘ moderate – change but not as fourfold or instructed
  - ∘ good – fourfold change and followed instructions
- In the usual care (no change) group:
  - ∘ poor – fourfold increase in maintenance dose
  - ∘ moderate – increase in maintenance dose but less than fourfold
  - ∘ good – no change in inhaled corticosteroid dose

We will also capture whether participants who have had a period of poor adherence simply restarted their inhaled corticosteroid on activation of zone 2.

### Outcomes

The primary outcome is ‘time to first asthma exacerbation’, defined as: the need for systemic corticosteroids and/or unscheduled health care consultation for asthma (i.e. reaching zone 3 or 4 of the Asthma UK self-management plan).

Secondary outcomes include the use of systemic corticosteroids and unscheduled health care consultations for an acute exacerbation of asthma:.total number of courses of systemic corticosteroids, total number of unscheduled health care consultations, time to participants requiring systemic corticosteroids and time to unscheduled health care consultation for an acute exacerbation of asthma, cumulative dose of inhaled and systemic corticosteroids used in the 12 months after randomisation, area under the morning peak flow curve over 2 weeks after activating stage 2 of the self-management plan and the Juniper Mini Asthma Control Questionnaire (MiniAQLQ) [9]. The cost and resource audits of both trial arms will be reported as incremental cost per asthma exacerbation prevented and cost per quality-adjusted life year (QALY) gained.

#### Participant timeline

The first patient was randomised on 17 May 2013 and recruitment will close on 31 January 2016 with the last patient’s last visit in January 2017.

Follow-up is at 6 months and at 12 months, either by clinic visit or by telephone. Participants are enrolled into the study for a total of 12 months. Information is being collected on orally administered corticosteroid use, unscheduled health care consultations, adverse events, EQ-5D-3 L and Service Use Questionnaire (SUQ) data. If asthma control deteriorates and zone 2 of the asthma plan is activated, an additional post-activation visit is required after the 14-day Asthma Diary Card has been completed. Adherence rating to the allocated asthma self-management plan (via research nurse review of completed Asthma Diary Card), exacerbation history and MiniAQLQ [9] data are also being collected at this visit Fig. 1.

**Table 1:** Summary of protocol amendments that impacted on trial design

Protocol	Date	Summary of protocol changes
V 2.0	5 Aug 2013	• Minor wording added to the protocol to clarify that potential participants can be recruited from clinic appointments in primary care
V 3.0	23 Oct 2013	• *Table 3 – How to achieve a quadrupling dose for participants on a combination inhaler* • Clarification of the dose strength for Symbicort • Clarification of inhaler type • The addition of a new dose of Fostair and QVAR • Clarification of a typographic error
V 4.0	3 Feb 2014	• Clarification of serious adverse event reporting timeframe
V 5.0	14 Oct 2014	• Use of the current approved advert to be placed on public notice boards, universities and on websites and social media • Use of DocMail in GP practices • Telephone consultation at 6 and 12 months if patient has not had an exacerbation
V 6.0	5 Nov 2015	• Additional wording to the sample size justification section of the protocol, reduction of sample size from 2300 to between 1774 and 1850, removal of paragraph and references pertaining to electronic dose counters (Smart-inhalers) and other minor typographic clarifications

A monthly text message reminder for follow-up visits is sent to the participants if they have a mobile phone and consent to receive this.

Details of the data collection schedule are summarised in Fig. 1.

#### Sample size

A reduction of one third in the number of people requiring treatment with orally administered corticosteroids was considered an important treatment effect from a group of local general practitioners, asthma nurses and asthma experts.

With 1000 participants per group, a log-rank test (at the two sided 5 % significance level) will have at least 90 % power to detect a difference of 30 %, assuming an exacerbation rate of 13 % in the control group. A 13 % exacerbation rate requiring systemic corticosteroids is the lowest level seen in the control group of previous studies of this type ([10, 11, 6]) and so provides us with a conservative estimate.

We initially proposed to recruit 2300 patients to allow for participants lost to follow-up, although we plan to obtain as much information about participants who have withdrawn as possible using computerised records.

The power calculation was revised in March 2015 in consultation with the NIHR Health Technology Assessment. The overall event rate in the first 226 participants recruited was shown to be higher around 20 % in those reaching the 12-month follow-up, and therefore the power calculation was re-calculated. Assuming a baseline exacerbation rate in the control group of 17 % and 90 % power, and still estimating a one third reduction in the four –fold increase group, then a sample size of 1542 is needed for analyses. Allowing for 20 % of participants being lost to follow up, we aim to recruit between 1774 and 1850 patients. Recruitment will close on 31st January 2016.

Due to the interim event rate for the primary outcome being higher than estimated the power calculation was revised. With a baseline exacerbation rate in the control of 17 % and 90 % power and still estimating a one-third reduction in the fourfold increase group, the sample size was revised to between 1750 and 1850 participants.

#### Recruitment

Potential participants are being recruited from both primary and secondary care in England and Scotland, with an estimated split of about 80:20, respectively.

Primary care recruitment is in general practices across England and Scotland in conjunction with Primary Care Research Networks (subsequently local Clinical Research Networks, CRN/SCRN), with practices either acting as Participant Identification Centres (PICs) or Research Initiative Sites (RIS). Participants are being identified by a database search and invitation letter and by opportunistic recruitment in RISs.

Secondary care recruitment is from respiratory outpatient clinics and via specific research volunteer databases.

#### Follow-up

Follow-up is at 6 months and at 12 months, either by clinic visit or by telephone. Information is being collected on orally administered corticosteroid use, unscheduled health care consultations, adverse events, the EuroQol five dimensions, three levels questionnaire (EQ-5D-3 L) and Service Use Questionnaire (SUQ) data. If asthma control deteriorates and zone 2 of the asthma plan is activated, an additional post-activation visit is required after the 14-day Asthma Diary Card has been completed. Adherence rating to the allocated asthma self-management plan (via research nurse review of completed Asthma Diary Card), exacerbation history and MiniAQLQ [9] data are also being collected at this visit.

A monthly text message reminder for follow-up visits is sent to the participants if they have a mobile phone and consent to receive this.

#### Participant withdrawal and stopping criteria

For individual participants, discontinuation of the treatment plan will be decided on an individual basis that will include, but not be limited to, people who have more than six asthma exacerbations in the 12-month trial period.

Adherence with the self-management plans will be reviewed at 12 months (and at 18 months if insufficient data is available). If the self-reported adherence is reliable, the futility analysis will be based on the entire study population. The Trial Steering Committee (TSC) will determine whether the overall adherence with the self-management plans makes the study futile and, therefore, should be stopped.

## Assignment of interventions

### Randomisation and blinding

The randomisation schedule is based on a computer-generated pseudo-random code using random permuted blocks of randomly varying size, created by the Nottingham Clinical Trials Unit (Nottingham CTU) in accordance with their standard operating procedure (SOP) and held on a secure University of Nottingham server. Randomisation is stratified by recruiting site (regional centre), smoking status (yes/no) and maintenance of inhaled corticosteroid dose (Appendix 5).

Investigators and research nurses access the randomisation website by means of a remote, Internet-based randomisation system developed and maintained by the Nottingham CTU. The sequence of treatment allocations will be concealed until interventions have all been assigned and recruitment, data collection and all other trial-related assessments are complete.

This is an open-label clinical trial, so the participant and the study team will be aware of the self-management plan allocation. The chief investigator and trial statisticians are blinded to participant treatment arms throughout the study. Participants will be enrolled and randomised by a member of the site study team.

## Data collection, management and analysis

### Data management

Clinic data are entered directly into a web-based trial database at the investigator sites by site users with unique login details. Patient questionnaires and Asthma Diary Cards completed at clinic visits are entered by the site nurses directly into the trial database.

Data quality is ensured by database validation checks which include missing data, out of range values, illogical entries and invalid responses. Data entered by sites into the trial database are subject to review by coordinating centre staff, and data queries are raised as necessary.

Detailed data management processes and procedures are documented in the FAST Data Management Plan.

### Statistical methods

A detailed statistical analysis plan has been agreed with the TSC. In summary, baseline characteristics will be described for both treatment groups. For the primary outcome variables, a log-rank test on Kaplan-Meier survival graphs for time to asthma exacerbation will be calculated. Multivariate analyses will also be investigated, using Cox regression to adjust for the effect of any potential differences between the groups at baseline. All analysis will be done as intention-to-treat. Subgroup analyses for smoking status at trial entry and high/low inhaled corticosteroid use at trial entry will also be performed.

## Monitoring

### Data monitoring

Integrity of trial conduct is overseen by the TSC, which meets at least once a year and provides overall supervision of the trial on behalf of the trial sponsor (University of Nottingham).

The Trial Management Group (TMG) meets more frequently and is responsible for the day-to-day management of the trial. Members of the TMG report to the TSC at their annual meetings.

The Data Monitoring Committee (DMC), meets at least once a year and provides independent oversight of trial data. The DMC report to the TSC.

The chief investigator has overall responsibility for the study and is custodian of the data.

### Interim analyses

There are no planned interim between-group analyses. However, progress with recruitment and retention is monitored monthly by the TMG. If progress is below target, strategies will be implemented to improve progress in discussion with the TSC.

### Assessment of harm

Adverse events are only reported during the 14 days following activation of zone 2 of the self-management plan and are limited to oral candidiasis and dysphonia as the adverse effects of inhaled corticosteroids are well-known.

Serious adverse events (SAEs) are only reported during the 14-day active treatment period, i.e. during activation of zone 2 of the self-management plan. Additionally any SAE that meets the criteria of a diagnosis of pneumonia is also recorded and reported up to 1 month after the 14-day activation period.

## Ethics and dissemination

### Research ethics approval

Ethics approval was granted by NHS Health Research Authority, North West – Greater Manchester South Research Ethics Committee and the respective National Health Service (NHS) Research and Development (R&D) departments for participating sites. The trial is being conducted in accordance with the ethical principles that have their origin in the Declaration of Helsinki, 1996; the principles of Good Clinical Practice and the Department of Health Research Governance Framework for Health and Social care, 2005.

### Protocol amendments

The methods described in this protocol reflect the current protocol (version 6.0 dated 5 November 2015). A summary of protocol amendments are summarised (Table 1).

All amendments to the protocol and associated paperwork have been approved by the trial sponsor, the Research Ethics Committee, local R&D departments and the trial funder prior to implementation.

### Consent

Written informed consent is obtained from all participants, who then provide demographic data on smoking history and baseline inhaled corticosteroid medication. Baseline peak expiratory flow (PEF), the MiniAQLQ [9] and the EQ-5D-3 L [www.euroqol.org] questionnaire scores are recorded and all data captured via an electronic data capture system.

No trial-specific procedures are conducted before informed consent has been obtained, and participants are reminded that they may withdraw from the trial at any time without it affecting the quality of their future care. Participants will be made aware (via the Participant Information Sheet and Informed Consent Form) that should they withdraw the data collected to date cannot be erased and may still be used in the final project analysis.

### Confidentiality

Individual participant medical information obtained as a result of this study is confidential. Participant confidentiality will be ensured using identification code numbers to correspond to treatment data in the computer files. Patient-identifiable information is not stored by the NCTU or sponsor.

Medical information may be given to the participant’s medical team and all appropriate medical personnel responsible for the participant’s welfare.

### Access to data

Data generated as a result of this trial will be available for inspection on request by the participating physicians, the University of Nottingham representatives, the Research Ethics Committee, local R&D departments and the regulatory authorities.

Requests for access to the original anonymised dataset should be made to the chief investigator.

### Post-trial care

After completing the study participants will continue to receive their standard asthma care through the NHS in accordance with local practice. A summary of the trial results will be provided to parents if they have given consent for this.

### Dissemination policy

Results will be reported in full through the National Institute for Health Research Journal series (open access), as well as through peer-reviewed journals, patient newsletters and websites.

The final report will follow the Consolidated Standards of Reporting Trials (CONSORT) 2010 guideline as well as its extension to pragmatic trials.

### Patient and Public Involvement

Patients and members of the public have been actively involved in the design and conduct of the FAST trial throughout.

Full details of the extent of Patient and Public Involvement, and the impact that this has had on delivery of the trial, will be reported separately.

### Trial status

The FAST trial is ongoing. The first participant was randomised on 17 May 2013 and recruitment will close on 31 January 2016 with the last patient’s last visit taking place on January 2017. Data collection, analysis and write-up should be completed by the end of 2017.

## Figures and Tables

**Fig. 2:**
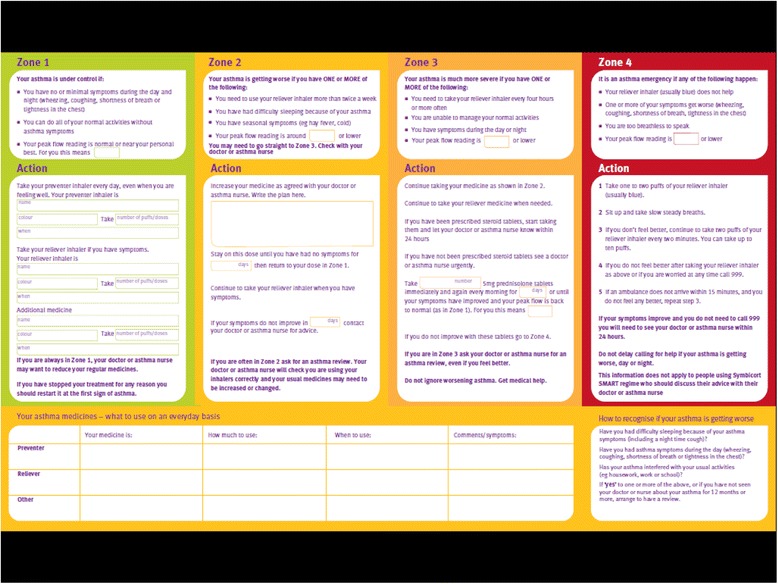
Asthma UK self-management plan

## Appendix 2

**Table 2:** Zone 2 of the asthma self-management plan

Active group	Control group
Zone 2	Zone 2
Your asthma is getting worse if you have one or more of the following: • You need your reliever inhaler more than twice a week • You had difficulty sleeping because of your asthma • You have seasonal symptoms (e.g. hay fever, cold) • Your peak flow is around ___ or lower	Your asthma is getting worse if you have one or more of the following: • You need your reliever inhaler more than twice a week • You had difficulty sleeping because of your asthma • You have seasonal symptoms (e.g. hay fever, cold) • Your peak flow is around ___ or lower
Action	Action
Use your reliever inhaler to relieve your symptoms and increase your preventer medication as described below *(individualised instructions on how to achieve a fourfold increase)* Once your symptoms or peak flow have returned to normal or after a maximum of 14 days return to your normal treatment If your symptoms get worse follow zone 3 instructions	Use your reliever inhaler to relieve your symptoms and continue your preventer medication at your normal dose Once your symptoms or peak flow have returned to normal or after a maximum of 14 days return to your normal treatment If your symptoms get worse follow zone 3 instructions
Start to record your morning peak flow, symptoms and medication in the study diary	Start to record your morning peak flow, symptoms and medication in the study diary
Phone your research nurse to arrange a study visit	Phone your research nurse to arrange a study visit

## Appendix 3

**Table 3:** How to achieve a quadrupling dose for participants on an inhaled corticosteroid-only inhaler (BDP,FP, budesonide, ciclesonide)

Current number of puffs	Puffs to achieve quadrupled dose
1 od	4 od
2 od	8 od
1 bd	4 bd
2 bd	8 bd

*bd* twice daily*, BDP* beclomethasone dipropionate, *FP* fluticasone propionate, *od* once daily

## Appendix 4

**Table 4:** How to achieve a quadrupling dose for participants on a combination inhaler

Current treatment	Additional treatment options	
	Option 1	Option 2
Seretide MDI 50/25 2 puffs bd	FP 50, 6 puffs bd	FP 125, 3 puffs bd
Seretide MDI 125/25, 2 puffs bd	FP 125, 6 puffs bd	FP 250, 3 puffs bd
Seretide MDI 250/25, 2 puffs bd	FP 250, 6 puffs bd	N/A
Seretide Accuhaler 100/50 1 puff bd	FP Disk 100, 3 puffs bd	N/A
Seretide Accuhaler 250/50 1 puff bd	FP Disk 250, 3 puffs bd	N/A
Seretide Accuhaler 500/50 1 puff bd	FP Diskhaler 500, 3 puffs bd	N/A
Symbicort Turbo 100/6 1 puff bd	Bud Turbo 100, 3 puffs bd	N/A
Symbicort Turbo 100/6 2 puffs bd	Bud Turbo 100, 6 puffs bd	Bud Turbo 200, 3 puffs bd
Symbicort Turbo 200/6 1 puff bd	Bud Turbo 200, 3 puffs bd	N/A
Symbicort Turbo 200/6 2 puffs bd	Bud Turbo 200, 6 puffs bd	Bud Turbo 400, 3 puffs bd
Symbicort Turbo 200/6 4 puffs bd	Bud Turbo 400, 6 puffs bd	N/A
Symbicort Turbo 400/12 1 puff bd	Bud Turbo 400, 3 puffs bd	N/A
Symbicort Turbo 400/12 2 puffs bd	Bud Turbo 400, 6 puffs bd	N/A
Fostair MDI 100/6, 1 puff bd	QVAR MDI 100, 3 puffs bd	N/A
Fostair MDI 100/6, 2 puffs bd	QVAR MDI 100, 6 puffs bd	N/A
Flutiform MDI 50/5, 2 puff bd	FP MDI 50, 6 puffs bd	N/A
Flutiform MDI 125/5, 2 puffs bd	FP MDI 125, 6 puffs bd	FP MDI 250, 3 puffs bd
Flutiform MDI 250/10, 2 puffs bd	FP MDI 250, 6 puffs bd	N/A

*bd* twice daily, *Bud* budesonide, *FP* fluticasone dipropionate, *MDI* metered-dose inhaler

## Appendix 5

**Table 5:** High and low doses for stratification purposes

Corticosteroid	Device and formulation	Low dose mcg/day	High dose mcg/day
BDP	Non-proprietary	100–1000	>1000–2000
BDP	Clenil MDI	100–1000	>1000–2000
BDP	QVAR MDI	50–500	>500–800
Budesonide	MDI	100–1000	>1000–1600
Budesonide	Turbuhaler	100–800	>800–1600
Fluticasone propionate	MDI/Accuhaler	50–500	>500–2000
Ciclesonide	MDI	80–320	
Seretide	MDI/Accuhaler	50–500	>500–1000
Symbicort	Turbuhaler	100–800	>800–1600
Fostair	MDI	400	
Flutiform	MDI	50–500	>500–1000

*BPD* beclomethasone dipropionate, *MDI* metered-dose inhaler

## Additional file

Additional file 1: SPIRIT 2013 checklist: recommended items to address in a clinical trial protocol and related documents. (DOC 121 kb)

## Abbreviations

CRN: Clinical Research Network
FAST: FourFold Asthma Study
GP: General practitioner
MiniAQLQ: Juniper Mini Asthma Quality of Life Questionnaire
NCTU: Nottingham Clinical Trials Unit
NHS: National Health Service
NIHR: National Institute of Health Research
NRES: National Research Ethics Service
PIC: Patient Identification Centre
SAE: Serious adverse event
SCRN: Scottish Clinical Research Network
SUQ: Service Use Questionnaire
QALY: Quality-adjusted life year
